# Non-Mucormycetes Causes of Fungal Rhino Sinusitis With Periocular Swelling in COVID-19 With Delta Variant

**DOI:** 10.7759/cureus.28825

**Published:** 2022-09-06

**Authors:** Anita Ambasta, Rakhi Kusumesh, Kamlesh Rajpal, Shailesh Kumar, Vivek Singh

**Affiliations:** 1 Ophthalmology, Indira Gandhi Institute of Medical Sciences, Patna, IND; 2 Microbiology, Indira Gandhi Institute of Medical Sciences, Patna, IND

**Keywords:** non-mucormycetes, delta variant, covid-19, fungal rhino sinusitis, covid 19, penicillium, culvularia, aspergillus, fusarium

## Abstract

Objective: To highlight fungi other than mucormycetes as causative agents of rhinosinusitis with periocular swelling in coronavirus (COVID-19) infection caused by Delta variant of SARS-CoV-2 virus and identify the presenting features, risk factors, intervention, and outcomes.

Methods and analysis: A retrospective interventional study of 96 patients with fungal rhinosinusitis and periocular swelling was done in patients with concurrent or recovered COVID-19 infection with the Delta variant (B.1.617.2) of SARS-CoV-2 virus in India. All patients with mucormycetes infection were excluded. Clinical presentation, medical history, blood reports, and imaging were analyzed. Management was by intravenous (IV) liposomal amphotericin B and functional endoscopic sinus surgery (FESS) with paranasal sinus debridement. Limited orbital debridement with or without transcutaneous retrobulbar liposomal amphotericin B (TRAMB) was done in patients with orbital involvement. Postoperative antifungal therapy was decided on the basis of the causative fungi.

Results: Four cases of Aspergillus and one each of Fusarium, Curvularia, and Penicillium-associated fungal rhinosinusitis with periocular swelling were seen. Signs of orbital involvement on MRI were present in all four of them. Two of these showed partial third-nerve palsy while one case with aspergillosis suffered cavernous sinus thrombosis. Proptosis was not witnessed in any case. History of diabetes and use of steroids was seen in all patients. All patients had mild to moderate COVID-19 with oxygen supplementation needed in one. No mortality, acute vision loss, or exenteration took place.

Conclusion: Aspergillus, Fusarium, Curvularia, and Penicillium were non-mucormycetes causes of fungal rhinosinusitis with periocular swelling in COVID-19 infection with the Delta variant (B.1.617.2) of SARS COV-2 virus. Few cases showed orbital and intracranial involvement.

## Introduction

The severe acute respiratory syndrome coronavirus-2 (SARS-CoV-2) Delta variant (B.1.617.2) was the cause of coronavirus disease 2019 (COVID-19) infection and the second wave of the pandemic in India from March to May 2021 [1]. These patients became vulnerable to fungal infection (mycosis) because of the compromised immune system, associated comorbidities such as diabetes mellitus, decompensated pulmonary functions, and the use of immunosuppressive therapy for the management of moderate to severe cases [2]. Some of them later developed fungal rhino sinusitis with associated periocular swelling. Though the mucormycetes group of fungi was commonly implicated, there were instances of other fungal infections (non-mucormycetes) with apparently similar presentations [2]. However, first-line treatment of different fungi will vary in terms of the appropriate antifungals, and empirical treatments should be avoided as much as possible [3]. This study demonstrates the presenting features, risk factors, interventions, and outcomes of patients with fungal rhino sinusitis and periocular swelling that were attributed to non-mucormycetes fungi in recovered or concurrent COVID-19 with Delta variant. This article was posted as a preprint on Research Square on March 28, 2022 (https://www.researchsquare.com/article/rs-1319142/v1).

## Materials and methods

A retrospective interventional study was done on patients with proven fungal rhinosinusitis and periocular swelling in recovered or concurrent COVID-19 infection who presented between April and June 2021 [4]. The place of study was a tertiary hospital that was designated as a dedicated hospital for COVID-19 which later also managed suspected mucormycosis patients. Demographic and clinical data were collected with the consent of patients and with the approval of the Ethics Committee (159/IEC/IGIMS/2021). The declaration of Helsinki was adhered to in this study. Each patient was subjected to a complete history-taking, including ocular complaints and the presence of co-morbidities along with their treatment. 

**Figure 1:**
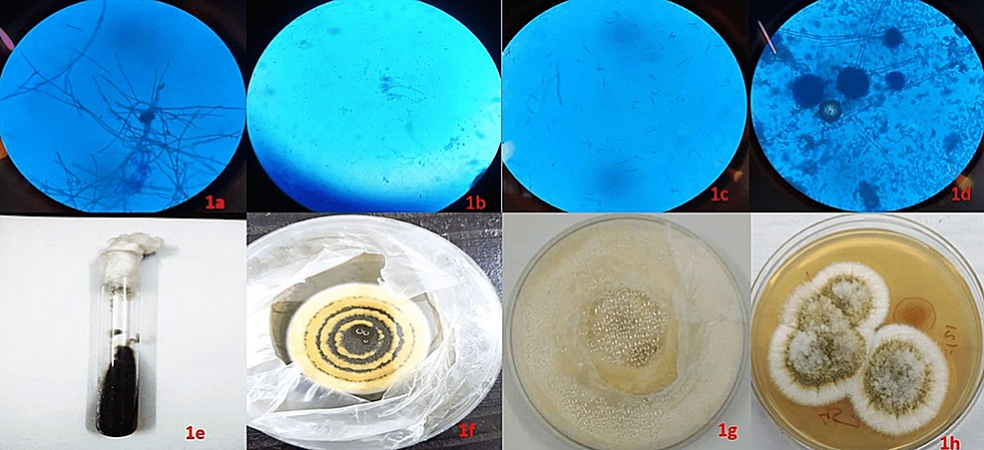
Microscopic images and fungal cultures of non-mucormycetes fungi in COVID-19 with Delta variant Microscopic images in lactophenol cotton blue mount (LPCB) of fungal isolates: (a) Curvularia sp (b) Penicillium sp (c) Fusarium sp (d) *Aspergillus flavus* Fungal culture done on Sabaraud dextrose agar showing: (e) Curvularia sp (f) Penicillium sp (g) Fusarium sp (h) *Aspergillus flavus*

COVID-19 diagnosis was based on real-time reverse transcription polymerase chain reaction (RT-PCR) testing using nasopharyngeal/oropharyngeal swabs. All or a few of the laboratory tests were performed in all cases: complete blood count (CBC), C-reactive protein (CRP), lactate dehydrogenase, procalcitonin, D-dimer, serum ferritin, and blood sugar. Magnetic resonance imaging (MRI) of the orbit, brain, and paranasal sinuses (PNS) with or without computed tomography (CT) was performed for assessing the extent of the disease. High-resolution computed tomography of the thorax and the COVID-19 reporting and data system score were used (RSNA consensus statement) to assess chest involvement, while the CT severity score was used to categorize patient disease as mild (<8), moderate (9-15), and severe (>15) [5]. Deep-seated tissue obtained with the help of a deep nasal swab or sinus endoscopy was sent for a KOH (potassium hydroxide) mount and fungal culture based on clinical suspicion. Proven mycosis was decided based on the former showing fungal hyphae in the biopsy specimen with associated tissue necrosis or a positive culture result done on specific fungal culture media such as Sabouraud dextrose agar [4]. Isolated fungal colonies were confirmed microscopically by performing a lactophenol cotton blue mount (LPCB) (Figure 1). Patients diagnosed with an infection from the mucormycetes group of fungi were excluded from the study. These were; Mucor, Rhizopus, Rhizomucor, Abidia, Apophysomyces, Saksenaea, and Cunninghumella [2]. The rest were referred to as non-mucormycetes fungi.

All the patients had been initiated on intravenous (IV) liposomal amphotericin B (5 mg/kg/day, up to a maximum of 10 mg/kg/day for CNS infections) with renal monitoring [6]. Functional endoscopic sinus surgery (FESS) with PNS debridement and liposomal amphotericin B irrigation was done by otolaryngologists in all cases who were RT-PCR-negative for COVID-19, and samples were sent for microscopic examination and fungal cultures. Limited orbital debridement with socket irrigation with amphotericin B was done in patients with radiological evidence of orbital involvement. In the interim waiting period, transcutaneous retrobulbar liposomal amphotericin B (TRAMB, 3.5 mg/mL) was administered in cases with ophthalmoplegia as part of another research project. Postoperatively, long-term antifungal treatment mostly with oral posaconazole (loading dose 300 mg twice a day on the first day, and maintenance dose 300 mg orally once a day, starting on the second day) was initiated, based on the microbiological report and drug availability. Other drugs such as oral voriconazole and itraconazole were also administered in a few cases.

## Results

Retrospective analysis of hospital records revealed that 96 patients with fungal rhino sinusitis and periocular swelling had been admitted during the study period. Seven patients out of these were determined to be due to non-mucormycetes fungi: four cases of Aspergillus (aspergillosis) and one each of Fusarium (fusariosis), Curvularia (curvulariosis), and Penicillium (penicilliosis) respectively. All patients had prominent periocular swelling and edema with nasal stuffiness (Table 1).

**Table 1:** Patient characteristics in seven non-mucormycetes fungal rhinosinusitis with periocular swelling in COVID-19 with delta variant ^a ^Day of mycosis detection after the onset of COVID -19 symptoms; ^b ^Para nasal sinus; ^c ^Functional endoscopic sinus surgery; ^d ^Transcutaneous retrobulbar liposomal amphotericin B; ^e ^Liposomal amphotericin B; ^f ^Treatment

Characteristics	Patient 1	Patient2	Patient 3	Patient 4	Patient 5	Patient 6	Patient 7
Fungi Identified	Curvularia	Aspergillus	Aspergillus	Aspergillus	Aspergillus	Penicillium	Fusarium
Age	47	52	66	57	34	33	45
Eye	LE	BL	LE	LE	BL	BL	LE
Sex	M	M	F	F	M	M	F
Day of onset^a^	15	20	18	27	21	6	17
CT severity Score	7	10	12	21	16	NA	NA
Periocular swelling	Present	Present	Present	Present	Present	Present	Present
Ophthalmoplegia	-	-	Present	-	-	Present	-
Vision	No loss	No loss	No loss	No loss	No loss	No loss	No loss
Oxygen support	None	None	None	Needed	None	None	None
Diabetes	Past history	New onset	Past history	Past history	New onset	New onset	New onset
PNS involved^b^	Sphenoid	Sphenoid	Maxilla	Maxilla	Maxilla	Diffuse	Maxilla
FESS^c^	Done	Done	Done	Done	Done	Done	Done
TRAMB^d^	-	-	Given	-	-	Given	-
L.AMPB^e^	Given	Given	Given	Given	Given	Given	Given
Other T/t^f^	Itraconazole	Voriconazole	Posaconazole	Posaconazole	Posaconazole	Posaconazole	Posaconazole

**Figure 2:**
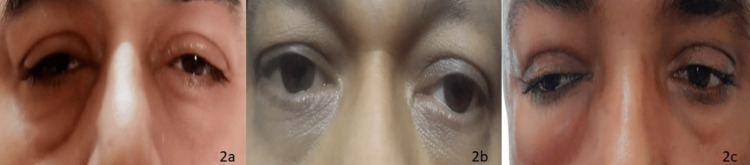
Bilateral periocular swelling in non-mucormycetes fungal rhinosinusitis in COVID-19 with delta variant (2a) patient 2, aspergillosis; (2b) patient 5, aspergillosis; (2c) patient 6, penicilliosis

Both eyes were involved in three cases (patients 2, 5, and 6) (Figure 2). There was no acute loss of vision in any of the seven cases. The ocular evaluation demonstrated partial third-nerve palsy with normal pupillary reactions in cases 3 and 6 (aspergillosis and penicilliosis, respectively). Proptosis was not witnessed in any case. Intracranial extension with cavernous sinus thrombosis was seen in one case with aspergillosis (patient 2). RT-PCR for COVID-19 was positive in five cases, while it was negative in two cases (patients 6 and 7). Case 6 was presumed to be a post-COVID-19 patient based on the clinical history and secondary fungal infection in the context of the raging pandemic. Case 7 had in all likelihood become RT-PCR negative due to a delay in presentation of symptoms of mycosis.

The geographic profile was limited to the Indian state of Bihar. The mean age of the patients at presentation was 47± 11 years. All patients showed a history of past- or recent-onset diabetes mellitus and had been administered IV dexamethasone during the disease course. No other underlying cause for immunosuppression was present. Mild to moderate COVID-19 was seen in all patients except patient 4, who needed oxygen support with a nasal prong (aspergillosis). Hematological investigations done at the time of presentation showed raised serum ferritin and CRP in all cases. Neutropenia was not present in any case. Lymphopenia was present only in case 6 (penicilliosis) (Table 2). This was also the only patient who complained of toothache and had the shortest interval (6 days) between the onset of COVID-19 symptoms and those of mycosis. There was no mortality or need for orbital exenteration in any case.

**Table 2:** Biochemical characteristics in seven non-mucormycetes fungal rhinosinusitis with periocular swelling in COVID-19 with delta variant ^a ^NA= Not Admissible. Normal range of parameters: ^b^Ferritin:20 to 250 ng/mL for adult males. 10 to 120 ng/mL for adult females; ^c^ D dimer: less than 0.50;  ^d ^C reactive protein: below 3.0 mg/L; ^e^ Lymphocyte:18-45% of total white blood cells; ^f^ Neutrophil: 40% to 60% of total white blood cells; ^g^ Total leucocyte count: 4,500 to 11,000 WBCs per microliter.

Parameters	Patient 1	Patient 2	Patient 3	Patient 4	Patient 5	Patient 6	Patient 7	Mean± SD
Fungi	Curvularia sp	Aspergillus flavus	Aspergillus flavus	Aspergillus Flavus	Aspergillus flavus	Penicillium sp	Fusarium sp	
Ferritin^b^	390	735	412	222	NAa	269	NA	405.6±200.7
D-Dimer^c^	2.3	1.31	1.51	0.97	0.28	0.41	NA	1.13±0.75
CRP^d^	120	50	24	15	20	NA	36	44.16±35
Lymphocyte^e^	16	30	13	9	10	18	18	16.28±1.41
Neutrophil^f^	76	64	81	78	84	76	76	76.4±6.26
TLC^g^	8630	9400	10400	12670	13020	4100	10540	9822±2986

Aspergillus was seen as septate hyphae branched at a 45° angle (patients 2, 3, 4, and 5) (Figures 1d, 1h). Diagnostic nasal endoscopy with biopsy from the affected side was inconclusive in three patients. However, all FESS specimens showed the presence of *Aspergillus flavus* in the fungal culture. All patients were put on oral posaconazole postoperatively. Along with FESS, limited orbital debridement and socket irrigation with liposomal amphotericin B was also done in case 2. However, this patient developed extensions into the cavernous sinus after discharge and was later administered an initial loading dose of IV voriconazole (6 mg/kg body weight) on day one followed by a maintenance dose of 4 mg/kg body weight for two weeks. Upon resolution of symptoms, a dosing regimen of 200 mg twice a day was followed. Patient 3 with ophthalmoplegia was given TRAMB while awaiting FESS.

Fusarium was seen as thin, septate, hyaline hyphae (patient 7) (Figures 1c, 1g). The patient presented 17 days after the onset of COVID-19 symptoms. An RT-PCR report was not accessible, and she was diagnosed with COVID-19 based on a high-resolution computed tomography (HRCT) report. Diagnostic nasal endoscopy with biopsy was confirmatory for Fusarium. The patient underwent FESS and sinus irrigation with liposomal amphotericin B followed by oral posaconazole (800 mg/24 h in two divided doses for 12-16 weeks).

Curvularia was seen as dematiaceous (dark pigmented) filamentous fungi (patient 1) (Figures 1a, 1e). The patient presented 15 days after the onset of COVID‑19 symptoms. Diagnostic nasal endoscopy and biopsy confirmed the presence of Curvularia. The patient underwent FESS and sinus irrigation with liposomal amphotericin B. The postoperative regimen included liposomal amphotericin B and itraconazole (200 mg twice orally).

Penicillium was seen as hyaline, septate hyphae on microscopic examination (patient 6) (Figures 1b, 1f). The patient presented with tooth pain, orbital and facial pain, and bilateral eye swelling. There was associated ophthalmoplegia in the same eye. Diagnostic nasal endoscopy was not confirmatory. TRAMB and FESS with limited orbital debridement and socket irrigation with liposomal amphotericin B were done based on MRI findings and clinical examination. Microscopic examination and the culture of the FESS specimen showed the presence of Penicillium sp. Postoperatively, oral posaconazole was prescribed. 

## Discussion

The Delta variant (or B.1.617.2 strain) of the coronavirus was primarily responsible for the second wave of COVID-19 in India from March to April 2021. It was 50% more transmissible than the Alpha variant (or B.1.1.7), with more severe disease and deaths [1,7]. The second wave also exhibited features such as headache, localized pain, nasal discharge, sinusitis, orbital cellulitis, and diminution of vision due to fungal rhinosinusitis with orbital or cerebral involvement occurring mainly after recovery from COVID-19. This was mostly attributed to the fungi mucormycetes [2]. Other fungi (mostly Aspergillus) have also been reported with a similar presentation in the second wave but at much less frequency. However similar reports in the context of COVID-19 were unavailable in literature for the fungi: Fusarium, Penicillium, and Curvularia. It is important to identify the causative fungi, as presentation might be similar but management protocol may differ, mainly in the use of appropriate antifungals. The conventional tests that identify a fungal pathogen and confirm a diagnosis of mycosis have different limitations, such as the time required to obtain a result or the impossibility of determining the fungus at the species level [8]. Species identification is also important for management which we were able to do in cases with aspergillosis (*Aspergillus flavus*). Hence antifungal treatment was initiated on the suspicion of possible mycosis without waiting for its confirmation, which would have meant a delay in initiating treatment and possible mortality. Another reason was that diagnostic nasal endoscopy with biopsy was deferred in patients who were not yet RT-PCR-negative due to the apparent risk of transmission of the virus to the health personnel. Treatment was subsequently reviewed and revised as per need according to the identity of the causative fungi.

In this study, we noted that Fusarium, Aspergillus, Penicillium, and Curvularia-associated rhinosinusitis with periocular swelling had similar presentations and risk factors. Case reports from pre-COVID times suggest that all of these can be associated with rhino-sino orbital disease [3,9-11]. Most of our patients had a history of mild to moderate COVID-19 disease, home treatment with no supplemental oxygen requirement, and recent-onset diabetes. The incidence of mycosis was hypothesized to increase considerably as a result of the widespread use of steroids, antibiotics, and antimetabolites in the second wave [12-14]. However, none of our patients gave a history of the latter. 

CT is a modality of choice when patient history and physical examination create suspicion of fungal infection, although MRI was predominantly used in our cases. MRI may be superior to CT for the evaluation of the orbital apex and cavernous sinus, and to detect enhancement of the optic nerve and cerebral dura [15]. Contrast-enhanced MRI showed maxillary sinusitis in the form of significant mucosal thickening to be predominant in most of our cases, although sphenoid was the predominant sinus involved in case 1 (Curvularia) and case 2 (aspergillosis). More than other fungi, Aspergillus has a predilection for affecting the sphenoid sinus-a finding that is poorly understood and may be related to low oxygen tension and a more acidotic environment within this sinus [15]. The disease’s invasive nature and the sphenoid’s proximity to critical structures of the skull base may explain the more aggressive clinical course in case 2 [16]. However, other cases of aspergillosis showed predominant maxillary sinusitis. Diffuse involvement of all sinuses was seen in penicilliosis.

Bilateral periocular swelling and edema with partial third-nerve palsy were seen in Aspergillus and Penicillium-associated rhinosinusitis (cases 3 and 6). The rest of the cases had periocular swelling and edema but no nerve palsy. Orbital involvement was seen as a hyperintensity of the retrobulbar space in MRI images with contrast in cases 2, 3, 6, and 7. Periocular swelling and edema in these cases may be attributed to the orbital involvement while in cases 1, 4, and 5 it may be a result of an inflammatory reaction to the fungal rhinosinusitis in the periocular tissue. There was no erythema or tenderness in any case; hence, cellulitis was ruled out. The partial third-nerve palsy in cases 3 and 6 resolved gradually over time. Many reports have suggested that cranial nerve palsy in the setting of COVID-19 infection may be associated with improved patient outcomes and temporary cranial nerve deficits compared to other neurological deficits, [17].

Aspergillus, a type of mold, was commonly seen in ICU-admitted patients causing pulmonary involvement in many studies in both coronavirus waves. However, there were only a few reports of Aspergillus rhinosinusitis with ocular findings. In our study, out of 96 patients with fungal rhino sinusitis and periocular swelling, we saw aspergillosis in four patients, with one developing intracranial extension on follow-up. A study by El-Kholy on fungal sinusitis in post-COVID-19 patients saw mucor species in 77.8% of cases and *Aspergillus fumigatus* in 30.6% [18]. One of the reasons for the predominance of mucormycosis in COVID-19 patients in India could be that it has a large population of people with diabetes mellitus, where it is predominantly seen [19]. Conversely, Aspergillus species are more common in other immunocompromised individuals in whom neutropenia is the dominant condition-especially persons with hematologic malignancies, hematopoietic stem cell transplant recipients, and solid-organ transplant recipients [20]. However, neutropenia was not present in any patient at presentation in our cases. Azoles such as voriconazole are the optimal choice if Aspergillus is proven on culture, although amphotericin is also active in many Aspergillus isolates [21]. In addition, newer azoles such as posaconazole and isavuconazole are promising secondary choices because they can be delivered orally and are thus an excellent choice for prolonged outpatient management [22].

Fusarium has been mentioned in the literature as the second-most-frequent mold after Aspergillus to be involved in fungal infections, especially among immunocompromised patients [23]. However, COVID-19-associated secondary infection has been dominated by molds such as mucormycetes. Even before the COVID-19 pandemic, the epidemiology of fungal infections among the immunocompromised patient population had been noted to have changed with the use of antifungal prophylaxis [24]. This change included a drop in the incidence of yeast infections, namely *Candida albicans*, and an increase in mold infections [24]. Successful treatment is highly dependent on the particular Fusarium species involved in the infection, which could not be ascertained in our case. However, our patient responded well to management by FESS and postoperatively to oral posaconazole. For the management of fusariosis, a high dose of IV amphotericin B formulation is recommended as the first line of therapy in patients. Voriconazole is also effective in treating fusariosis. Intolerance, contraindication, or failure of the amphotericin B formulation warrants the use of voriconazole as an alternative agent, and posaconazole is licensed as a salvage therapy against invasive fusariosis [25].

Penicillium is a mold and is usually considered a contaminant or colonizer, which can occasionally cause a life-threatening infection [10]. Our patient had raised markers of inflammation with new-onset diabetes and was the only patient in our series to have lymphopenia at the time of presentation. The reason for this could be that the time from onset of COVID-19 to symptoms of mycosis was the shortest with six days when the patient had probably not yet fully recovered from an infection, which is more associated with lymphopenia. According to one report, a shorter time duration between the onset of COVID-19 and symptoms of mucormycosis entails a more severe disease which may have been the case here too [26]. The patient showed orbital involvement, as seen by partial third-nerve palsy and the MRI report. We could not identify the species here, but few are highly susceptible to miconazole, itraconazole, ketoconazole, and 5-fluorocytosine. Amphotericin B shows intermediate antifungal activity, while fluconazole is the least active [27].

There are few reports of Curvularia-associated fungal rhino sinusitis, although CNS involvement has also been reported. A case series on human Curvularia infections by Rinaldi noted that none of his patients suffered from known immunologic disorders or underlying debilitating diseases; however, our patient had preexisting diabetes mellitus [28]. All patients in this case series had been treated by surgical management of sinus disease with variable use of antifungals and good outcomes similar to ours. The in vitro antifungal susceptibility of clinical isolates of Curvularia was tested against nine drugs using a reference microdilution method in a study and the most active drugs found were echinocandins, amphotericin B, and posaconazole while voriconazole and itraconazole showed poor activity [29].

To sum up, treatment of non-mucor fungal rhinosinusitis with periocular swelling included systemic antifungal agents, reversal of immune dysfunction, and FESS with optimal surgical resection of necrotic tissues if present. Theoretically, surgical therapy reduces the fungal burden, and the remainder of the fungi can be reached by systematic antifungal therapy. However, in many hospitals like ours where FESS was delayed until the patient became RT-PCR negative for COVID-19, antifungals, and TRAMB were administered for disease control in the interim period. The pandemic also saw a shortage of liposomal amphotericin B in most places and knowledge of the efficacy of other antifungals in different fungal infections can help in the continuation of treatment.

Limitations

Lack of histopathologic evidence of fungal invasion of tissues and vessels as well as the host reaction to the fungus. The study was limited to a single state.

## Conclusions

Fusarium, Aspergillus, Penicillium, and Curvularia were a few non-mucormycetes fungi that caused fungal rhinosinusitis with periocular swelling in the context of coronavirus-2 (SARS-CoV-2) infection with Delta variant (B.1.617.2) in India. Orbital or intracranial involvement was present in a few of the patients included in this study. There was no mortality or acute loss of sight. Timely initiation of appropriate anti-fungal therapy and surgical management limits the spread of the disease.
